# Integrated analysis of osmotic dehydration of bocaiuva (*Acrocomia aculeata*) slices

**DOI:** 10.1515/biol-2025-1271

**Published:** 2026-01-23

**Authors:** João Renato de Jesus Junqueira, Thiago Ferreira Rangel, Raquel Pires Campos, Thaísa Carvalho Volpe Balbinotti, Luciana Miyagusku, Jefferson Luiz Gomes Corrêa

**Affiliations:** Federal University of Minas Gerais/UFMG – Pharmacy Faculty, Av. Presidente Antônio Carlos, 6627, Campus Pampulha, 31270-901, Belo Horizonte, MG, Brazil; Federal University of Mato Grosso do Sul/UFMS – Faculty of Pharmaceutical Sciences, Food and Nutrition, Cidade Universitária, 79070-900, Campo Grande, MS, Brazil; Federal University of Lavras/UFLA – Department of Food Science, 37200-000, Lavras, MG, Brazil

**Keywords:** vacuum application, centrifugal force, principal component analysis, native fruit, mass transfer intensification

## Abstract

This study evaluated the effects of temperature, osmotic agent type, and process intensification techniques on the osmotic dehydration of bocaiuva (*Acrocomia aculeata*) slices. Treatments were conducted at 40 and 60 °C using sucrose or maltodextrin solutions, under static conditions as osmotic dehydration (OD), vacuum osmotic dehydration (VOD), or centrifugal osmotic dehydration (COD). Mass transfer parameters (water loss (WL), solid gain (SG), and moisture content) and quality indicators (shrinkage and rehydration) were determined. Sucrose-based treatments showed superior dehydration performance compared to maltodextrin, mainly under centrifugal force at 60 °C, which resulted in the highest WL (11.48 kg/100 kg) and the lowest moisture content (28.46 kg/100 kg), as well as reduced shrinkage and consistent rehydration capacity (0.885 and 0.93, respectively). Principal component analysis (PCA) confirmed the positive interaction between sucrose, elevated temperature, and COD, highlighting this combination as the most effective for improving dehydration efficiency while preserving physical quality. The findings contribute to the development of sustainable technologies that add value to native fruits, thereby reinforcing the role of food processing in promoting sociobiodiversity.

## 1 Introduction

The state of Mato Grosso do Sul (Brazil) presents a great diversity of native species, requiring techniques that support the valorization and preservation of its biomes, especially the Cerrado and Pantanal [1], [2], [3].

The palm tree *Acrocomia aculeata* (Jacq.) Lodd is native to the central region of Brazil, mainly occurring in the Cerrado and Pantanal biomes. The economic importance of this plant is attributed to the commercialization of its fruits, which are edible and serve as a source for oil extraction, in addition to playing a role in social identity and having environmental relevance [4], [5], [6].

In Brazil, the fruits of this palm are known as bocaiuva, macaúba, coco-de-catarro, chiclete-de-baiano, or spiny coconut, presenting nutritional potential as a natural source of vitamins, minerals, fibers, and bioactive compounds [4], 7].

Due to its seasonality and perishability, techniques that extend the shelf life of bocaiuva fruits while preserving their characteristics are necessary. Processes that reduce the moisture content of the fruits can be employed to obtain differentiated products with added value and increased stability [6], 8].

Osmotic dehydration (OD) is a process that consists of the partial removal of moisture from cellular tissues by the immersion of the food in a hypertonic solution. OD is a drying pre-treatment, used to maintain the sensory, physical, and nutritional quality of foods for subsequent processing stages, and is commonly applied to foods, especially fruits [9], [10], [11].

During OD, two mass transfer fluxes occur: moisture migrates from the food matrix to the hypertonic solution, while solutes diffuse from the solution into the tissue (impregnation) [12], [13], [14]. When mass transfer becomes limited, complementary techniques can be applied to promote microstructural changes that facilitate water removal and improve overall mass transfer efficiency [10].

The use of innovative OD techniques as pre-drying treatments offers promising strategies to improve product quality and reduce energy demand during subsequent dehydration steps. Their application in native fruit processing can add value to regional biodiversity and mitigate post-harvest losses, contributing to both economic viability and environmental sustainability.

The application of reduced pressure (vacuum osmotic dehydration – VOD) enhances mass transfer by combining osmotic pressure gradients with a vacuum-induced pressure differential. The process initiates a hydrodynamic mechanism that expels gases and liquids from cell pores, allowing osmotic solution penetration. This improves dehydration efficiency and enables controlled impregnation of active compounds [15], [16], [17], [18].

Centrifugal osmotic dehydration (COD) enhances mass transfer by applying centrifugal force during the initial stages of the process. This force accelerates moisture removal and reduces solute uptake by promoting the movement of liquid from the product to the solution. It improves dehydration efficiency and shortens processing time [10], 19], 20].

Variables such as temperature, type, and concentration of the osmotic agent influence mass transfer parameters and the final quality of the product. The choice of solute is crucial, as it influences sensory properties, nutritional value, and processing cost. Its molecular weight and ionic behavior significantly affect water loss (WL) and solid gain (SG) during osmotic dehydration [12], 21], 22].

Therefore, this study aimed to evaluate the effects of different OD temperatures (40 and 60 °C) and the use of different osmotic agents (sucrose and maltodextrin) on the mass transfer parameters of osmotically dehydrated bocaiuva slices. The effectiveness of applying vacuum (VOD) and centrifugal force (COD) in the first minutes of the process was also assessed in a univariate/multivariate statistical approach.

## 2 Materials and methods

### 2.1 Materials

The bocaiuva fruits (*A. aculeata*) used in the OD experiments were harvested from the Cerrado region (20°28′11″ S, 54°37′12″ W) in Mato Grosso do Sul, Brazil. Only fully mature fruits, visually free of injuries, mechanical damage, or microbial deterioration, and presenting similar size and weight were selected to ensure experimental uniformity [6].

After manual selection, washing, and sanitization, the epicarp and endocarp were removed. Mesocarp slices were prepared and stored at −18 °C until use. Before processing, samples were thawed under refrigeration at 4 °C for 12 h and then equilibrated at room temperature.

The initial moisture content was 40.1 ± 1.5 kg water/100 kg sample (wet basis), determined by oven drying at 105 °C until a constant weight was achieved [23]. Physicochemical characterization of the fruit included measurements of pH (HANNA pH21 pH meter, Brazil), total soluble solids (Abbè refractometer, Tecnal RL3, Brazil) and titrable acidity (titration with a standard solution of sodium hydroxide (0.1 M) and phenolphthalein at 1 % as an indicator) [24]. The pH value was 6.46 ± 0.07, the soluble solids content was 2.6 ± 0.2 ° Brix and the titrable acidity was 0.17 ± 0.01 g_citric acid_/100 g.

### 2.2 Sample and osmotic solution preparation

The sample slices were obtained with the aid of a stainless steel knife in a parallelepiped shape (2.00 ± 0.05 cm length, 0.75 ± 0.05 cm width, 0.25 ± 0.05 cm thickness) [25]. Binary osmotic solutions were prepared with distilled water at a concentration of 60 kg_solute_/100 kg_solution_. Sucrose and maltodextrin (ED 10) were used as osmotic agents.

### 2.3 Osmotic dehydration (OD)

The effects of temperature and different osmotic agents were evaluated using a full 2 × 2 factorial experimental design, considering two types of osmotic agents (sucrose and maltodextrin) and two processing temperatures (40 °C and 60 °C).

The samples were placed in 25 mL flasks containing the osmotic solution at a solution-to-sample ratio of 20:1 (w/w). The temperature was maintained at a constant level using a thermostatic water bath, with fluctuations of ± 2 °C. The total processing time was 120 min, a period during which the highest mass transfer rates are typically observed in dehydration processes [26].

After this period, the samples were removed from the solution and immersed in cold water for 10 s to halt the osmotic process. Each sample was gently blotted with absorbent paper to remove surface solution. Samples were then weighed and their moisture content determined by oven drying at 105 °C until a constant weight was achieved [23].

All experiments were performed in triplicate, and the mean values were reported. Equations (1) and (2) were used to calculate water loss (WL) and solid gain (SG), respectively [9], 26].

(1) $$\text{WL}=\frac{\left(M_{0}X_{w0}\right)-\left(M_{f}X_{\text{wf}}\right)}{M_{0}}$$

(2) $$\text{SG}=\frac{\left(M_{f}X_{\text{sf}}\right)-M_{0}\left(1-X_{w0}\right)}{M_{0}}$$

where  *M*_0_ is the weight of the sample at time t = 0 s [kg], *X*_*w*0_ is the initial water content [kg water 100/kg sample], *M*_*f*_ is the weight of the sample at time t = 120 min [kg], *X*_wf_ is the water content [kg water 100/kg sample] at time t = 120 min and *X*_sf_ is the soluble solid content [kg solid 100/kg sample] at time t = 120 min.

### 2.4 Osmotic dehydration: application of vacuum (VOD) and centrifugal force (COD)

The selection of intensified experimental conditions followed a hierarchical experimental design approach, as described in Section 2.3. A reference operational condition was first established, and this condition was subsequently used for the following process intensification techniques.

The vacuum-assisted treatments (VOD) were carried out with a vacuum pressure of 5.333 × 10^4^ Pa, applied during the first 10 min of the process [27], using a vacuum pump (Prismatec, model 131, Brazil). After that, atmospheric pressure (1.011 × 10^5^ Pa) was restored and maintained throughout the process.

The centrifugal force application (COD) was tested by placing the samples in tubes in a centrifuge (Centribio, model 80-2B, Brazil) at a rotational speed of 2,800×g [19].

### 2.5 Physical analyses

The volumetric shrinkage was determined by measuring the surface area and thickness of the samples. The surface area was determined through digital image analysis using the free software ImageJ (version 1.45s), which converts pixel data into real-dimensional values based on a calibrated reference scale [17].

The sample thickness was measured at five distinct points using a digital caliper (Vonder, 150 mm, with a precision of ± 0.01 mm). The volumetric shrinkage was calculated as the ratio between the initial volume [m^3^] and the volume after 120 min [m^3^]. Higher values indicate a lower degree of shrinkage [17].

Rehydration was determined by immersing the osmodehydrated slices in distilled water at room temperature (25 ± 1 °C) for 20 h. The immersion time was selected to ensure that the samples approached hydration equilibrium under the experimental conditions [20]. The mass ratio between the samples and the distilled water was 1:25. The weight change was calculated after gently removing excess surface water using absorbent paper. The rehydration was calculated as the ratio between the weight of the dried sample and the weight of the fresh sample [6], 20].

### 2.6 Statistical and multivariate analysis

Normality and homogeneity of variances were verified using the Shapiro–Wilk and Levene’s tests, respectively, before analysis of variance (ANOVA). All experimental data were initially subjected to a one-way analysis of variance (ANOVA) to evaluate the effects of temperature, osmotic agent, and process intensification technique on technological parameters (WL, SG, shrinkage, rehydration, and final moisture content). When significant differences were detected (*p* < 0.05), Tukey’s multiple comparison test was applied. These tests were performed using Sisvar software (version 5.6, Brazil) [28].

To complement the univariate approach, a multivariate statistical analysis was performed using Python 3.11 and the libraries Pandas, Statsmodels, Seaborn, Scikit-learn, and Matplotlib.

Pearson’s correlation matrix was constructed to assess linear relationships between technological parameters and to identify redundancy or trade-offs among responses. The correlation coefficient (r) was interpreted as: weak (|r| < 0.3), moderate (0.3 ≤ |r| < 0.7), or strong (|r| ≥ 0.7) [29].

A Principal Component Analysis (PCA) was conducted on the normalized data to identify patterns of similarity or divergence between treatments and reduce data dimensionality. The first two principal components (PC1 and PC2) were plotted to visualize treatment distribution in multivariate space and to interpret which variables most influenced clustering [30].

## 3 Results and discussion

### 3.1 Influence of temperature and osmotic agents

**Figure 1:**
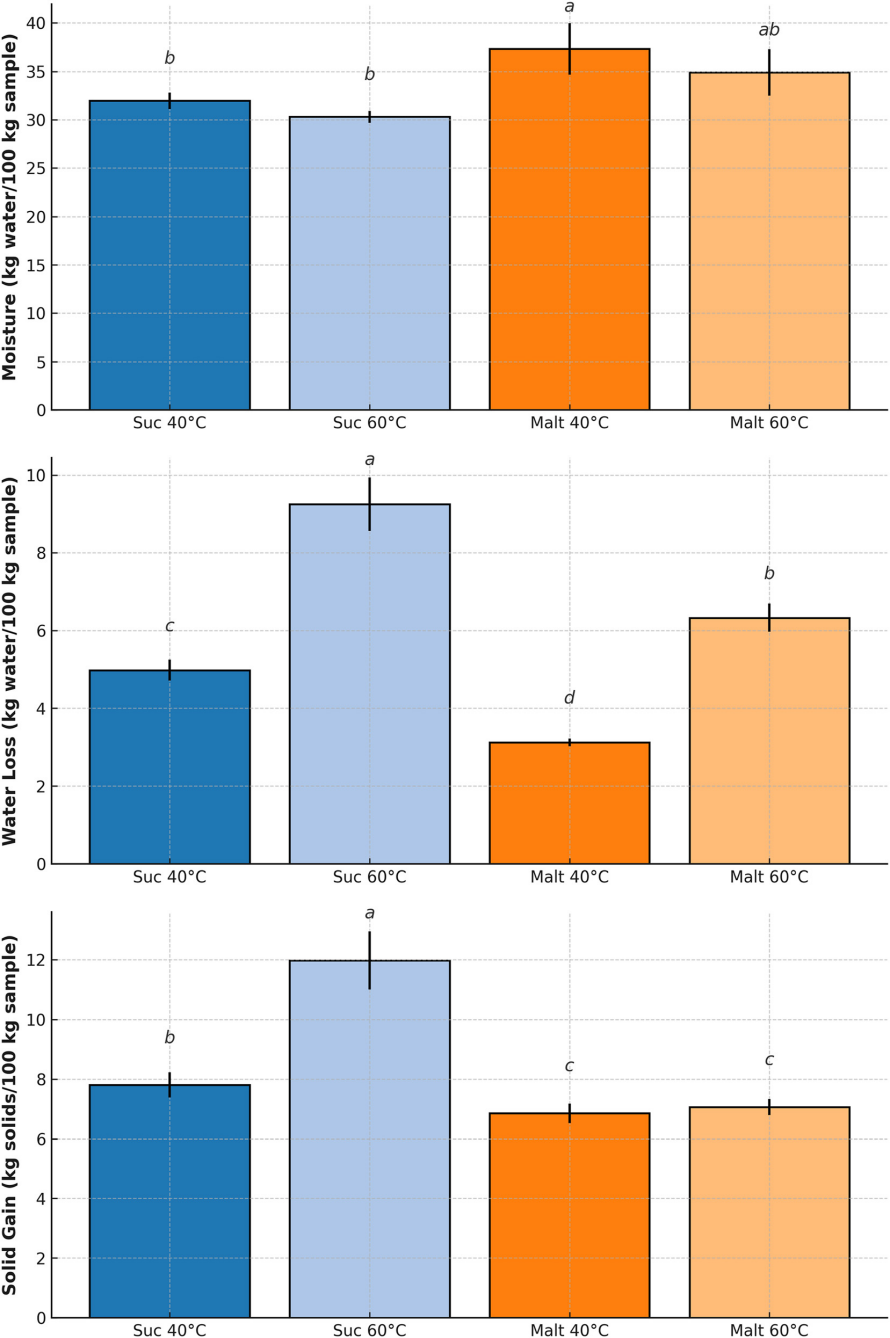
Moisture content, water loss (WL), and solid gain (SG) of osmotically dehydrated bocaiuva slices treated with sucrose (Suc) or maltodextrin (Malt) at 40 and 60 °C. Means followed by the same letter do not differ significantly according to Tukey’s test (*p* > 0.05).

Figure 1 shows the influence of both temperature (40 and 60 °C) and osmotic agent (sucrose–Suc or maltodextrin–Malt) on the mass transfer parameters, including moisture content, WL, and SG, of the osmotically dehydrated bocaiuva.

Significant differences were observed between temperatures (*p* ≤ 0.05) for all the evaluated variables. The highest moisture contents were recorded in samples treated with maltodextrin (Figure 1), with the treatment at 40 °C differing statistically from the others.

As presented in Figure 1, the increase in temperature from 40 °C to 60 °C significantly reduced the moisture content in bocaiuva slices, providing enhanced WL at higher temperatures. The combination of sucrose and 60 °C resulted in the lowest moisture content, while maltodextrin at 40 °C led to the highest (*p* ≤ 0.05).

The increase in osmotic solution temperature is associated with an increase in cell membrane permeability and a reduction in the viscosity of the solution. Although temperature did not markedly influence the moisture content in some treatments, it enhanced WL and SG by decreasing internal and external resistances to mass transfer [31].

Both moisture and solute diffusivities increase with temperature [18]. Since OD is inherently a partial dehydration process, such increases directly accelerate water removal and solute incorporation [32], 33].

A similar behavior was observed during the OD of melon [34], 35], carrots [36], and pineapple [37] in osmotic solutions. Those authors reported that higher WL and SG were obtained at higher temperatures.

Regarding the osmotic agent employed, sucrose solution generally led to greater moisture reduction in the osmotically dehydrated product and promoted higher WL and SG rates (Figure 1). The highest WL was observed in the treatment carried out at 60 °C with sucrose (9.25 ± 0.69 kg/100 kg), which differed significantly from the other treatments (*p* ≤ 0.05).

Sucrose (C_12_H_22_O_11_, 342.29 g/mol) presents a lower molar mass than maltodextrin [(C_6_H_10_O_5_)_n_, with 2 < *n* < 20]. Higher molar mass solutes tend to yield osmotic solutions with higher water activity [38]. Since chemical potential gradients drive mass transfer, the greater the difference in water activity between the product and the solution, the more efficient the dehydration process occurs [10].

Due to its lower molar mass, sucrose promotes a greater reduction in solution water activity and consequently in the water activity, compared to maltodextrin. It also generates higher osmotic pressure, which explains the higher mass transfer values obtained when using sucrose [10]. As the solute is incorporated into the food, it becomes linked to the food’s moisture, making this moisture unavailable and decreasing water activity [32], 39].

Similarly, some authors reported higher mass transfer rates in yacon slices osmodehydrated in solutions with lower molar mass solutes [40], 41]. From a dehydration perspective, treatments that enhance WL are desirable. Therefore, based on the results in Figure 1, the temperature of 60 °C was selected for the subsequent osmotic dehydration experiments, as it resulted in the most favorable mass transfer conditions.

### 3.2 Influence of vacuum and centrifugal force application

The effect of applying different techniques during the osmotic dehydration of bocaiuva slices at 60 °C was evaluated. For both tested solutions, the vacuum was evaluated using vacuum osmotic dehydration (VOD), and the centrifugal force was assessed in centrifugal osmotic dehydration (COD).

**Figure 2:**
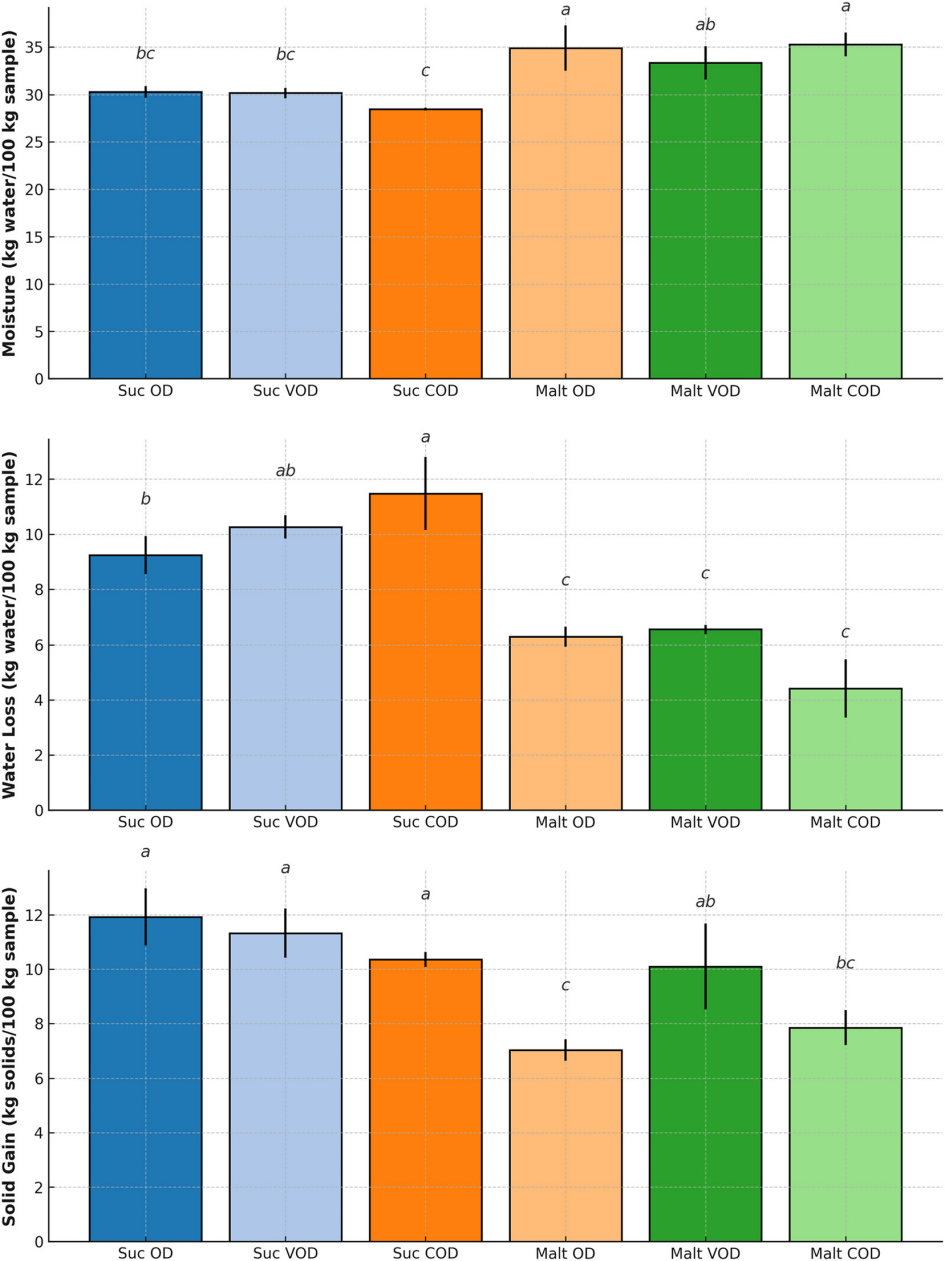
Mass transfer parameters of bocaiuva slices subjected to osmotic dehydration (OD), vacuum osmotic dehydration (VOD), and centrifugal osmotic dehydration (COD) with sucrose (Suc) or maltodextrin (Malt). Means followed by the same letter do not differ significantly according to Tukey’s test (*p* > 0.05).

The results for moisture content, WL, and SG are presented in Figure 2. Significant differences (*p* ≤ 0.05) were found for all variables among the different treatments.

Lower moisture contents were observed in the treatments using sucrose as the osmotic agent, with no significant differences between the treatments (*p* > 0.05). Besides, higher moisture contents were recorded in the bocaiuva slices osmotically dehydrated in maltodextrin solutions (Figure 2), but again with no significant differences observed among the treatments.

According to Figure 2, higher WL rates were observed for osmotic processes using a sucrose solution, both with vacuum (VOD) and centrifugal (COD) treatments.

When reduced pressure is applied in the first minutes of OD, occluded gases within the porous structure of the food expand and are subsequently removed. Once atmospheric pressure is restored, the empty spaces are filled with osmotic solutions, thereby increasing the specific surface area for mass transfer [39], 42].

The effectiveness of vacuum application depends on several factors, particularly the porosity and microstructural arrangement of the food matrix. In general, the higher the porosity of the food matrix, the greater the influence of vacuum on mass transfer phenomena [17].

Higher WL values were observed during the VOD of papaya slices compared to conventional OD [39]. Similar results were reported when comparing VOD with OD for mango and yacon slices [15], 33].

The application of centrifugal force (COD) also resulted in higher WL rates (11.48 ± 1.32 kg/100 kg sample) when sucrose was used as the osmotic solution (Figure 2). Similar results were found during osmotic processes applied to carambola slices [19]. Those authors reported higher WL in treatments subjected to centrifugal force, compared to those carried out with OD.

Recent reviews have observed that centrifugal force can significantly enhance osmotic dehydration efficiency by reducing moisture content without requiring a phase change in water [10], corroborating the present work.

Centrifugation assists in the removal of intracellular water through osmosis, thereby increasing the chemical potential gradient relative to the solution and enhancing WL [20].

In samples treated with maltodextrin solutions, no noticeable improvements in WL were observed, probably due to the higher viscosity of the osmotic solution, which may restrict the effects of vacuum or centrifugal force application during dehydration. The impact of the different intensification techniques on SG could not be clearly distinguished among treatments (Figure 2).

In osmotic dehydration, solutes with higher molar mass tend to exhibit lower incorporation into the product. Moreover, the high viscosity of the maltodextrin solution tends to form a thin barrier layer on the fruit surface, hindering both water removal and solute diffusion. Consequently, samples show higher moisture content and lower WL and SG values [10].

On the other hand, samples treated with sucrose solution exhibited higher SG values, as the lower molar mass of sucrose favors solute diffusion and enhances mass transfer rates [43].

**Figure 3:**
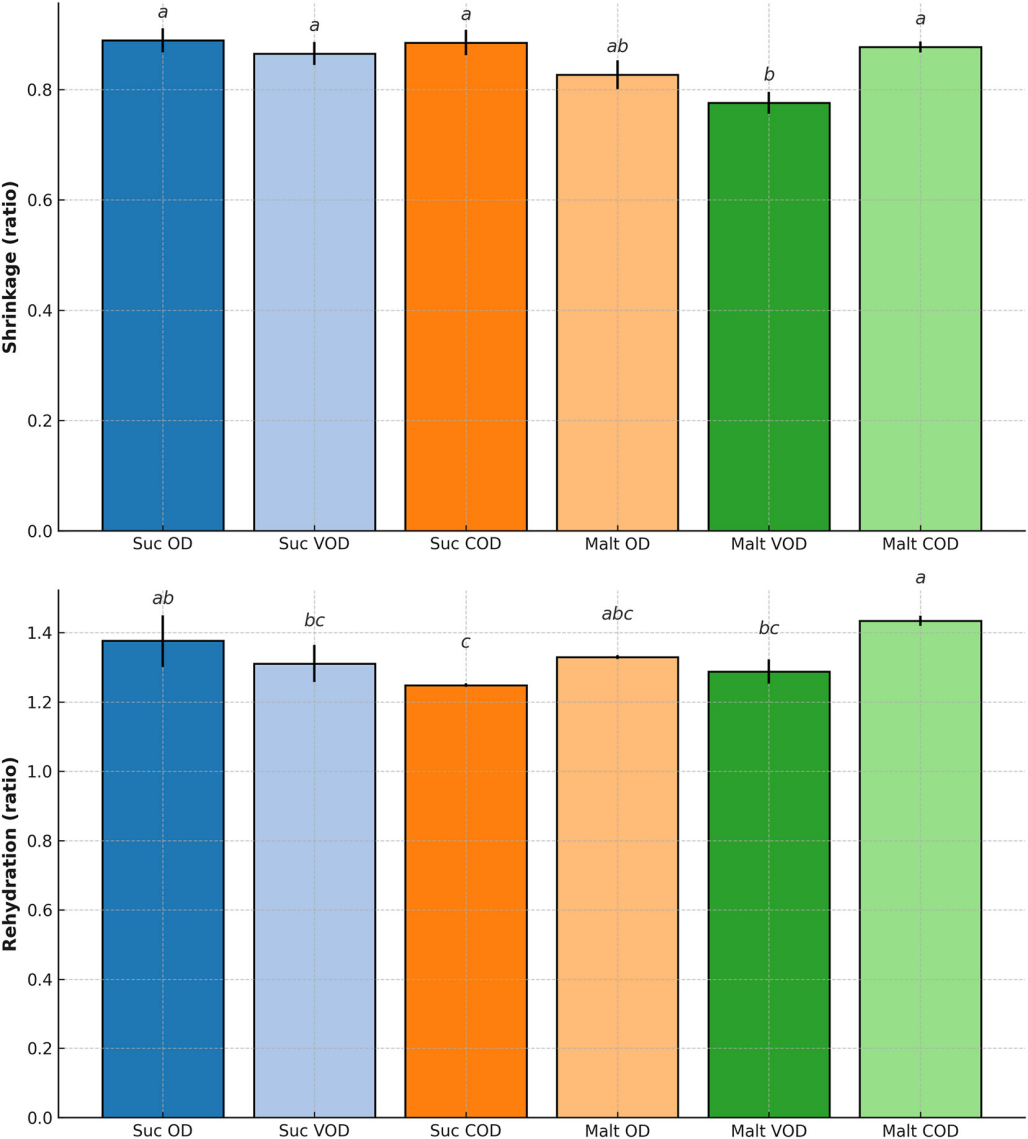
Shrinkage and rehydration capacity of osmotically dehydrated bocaiuva slices treated with sucrose or maltodextrin solutions under osmotic dehydration (OD), vacuum osmotic dehydration (VOD), and centrifugal osmotic dehydration (COD). Means followed by the same letter do not differ significantly according to Tukey’s test (*p* > 0.05).

For the different treatments, the mean values (and standard deviations) of shrinkage (expressed as the volume ratio) and rehydration rate are presented in Figure 3. Significant differences (*p* ≤ 0.05) were observed among treatments in these results.

Volumetric shrinkage values closer to one indicate lower dimensional changes, which may be indicative of improved physical stability of the product. These changes can be tentatively related to tissue-level modifications promoted by the dehydration process [38], 44].

The reduction in sample dimensions is a parameter directly associated with food shrinkage during dehydration processes. It is observed that a reduction in the moisture content may be associated with changes in the mechanical tension exerted by water within the cellular structure. This may create a pressure imbalance between the internal and external environments of the tissue, potentially causing structural rupture and collapse [45], 46].

Macedo et al. [13] observed that papaya slice subjected to OD and VOD processes with isomaltulose as an osmotic agent had their volume shrinkage levels ranging from 11 to 20 %. Shrinkage during osmotic processes was also reported in beetroot, eggplant, and carrot slices subjected to OD and VOD, in varying ranges depending on the vegetable type [17].

Yang et al. [46] observed that fresh blueberries subjected to OD with xylitol as an osmotic agent had their volume shrinkage levels ranging from 6 to 8 %. Macedo et al. [47] observed that the type of osmotic agent did not influence the shrinkage of strawberry cubes subjected to OD using sucrose and coconut sugar.

The samples submitted to all the osmotic processes were able to be rehydrated (Figure 3), as the rehydration ratio was higher than one. However, it was not possible to clearly distinguish the effect of each intensification technique on this parameter.

Treatments that promoted lower mass transfer resistance during dehydration (VOD and COD) tended to exhibit higher rehydration ratios, indicating a more favorable physical configuration of the food matrix. Although rehydration performance is often associated with tissue integrity, the present results were interpreted cautiously, as no direct microstructural analyses were performed [48], 49].

In general, the rehydration behavior of osmotically dehydrated bocaiuva slices followed the same trend reported for other osmotic treatments. Shrimp subjected to OD packaging showed a clear dependence of rehydration capacity on process conditions and structural changes of the muscle tissue [50].

Similarly, Fadeyibi et al. [51] observed that dried okra slices processed by OD combined with non-ionizing radiation reached a maximum rehydration ratio of approximately 2, without fully recovering the original structure, due to cell wall collapse and reduced porosity.

In our study, the rehydration behavior of bocaiuva slices was comparable to that reported for other osmotically dehydrated products, indicating that mass transfer intensification did not severely compromise water uptake capacity.

### 3.3 Multivariate approach

To provide an integrated interpretation of the experimental data, multivariate statistical techniques were applied to evaluate the interdependence between technological parameters and to characterize the performance of each treatment in a multidimensional space.

**Figure 4:**
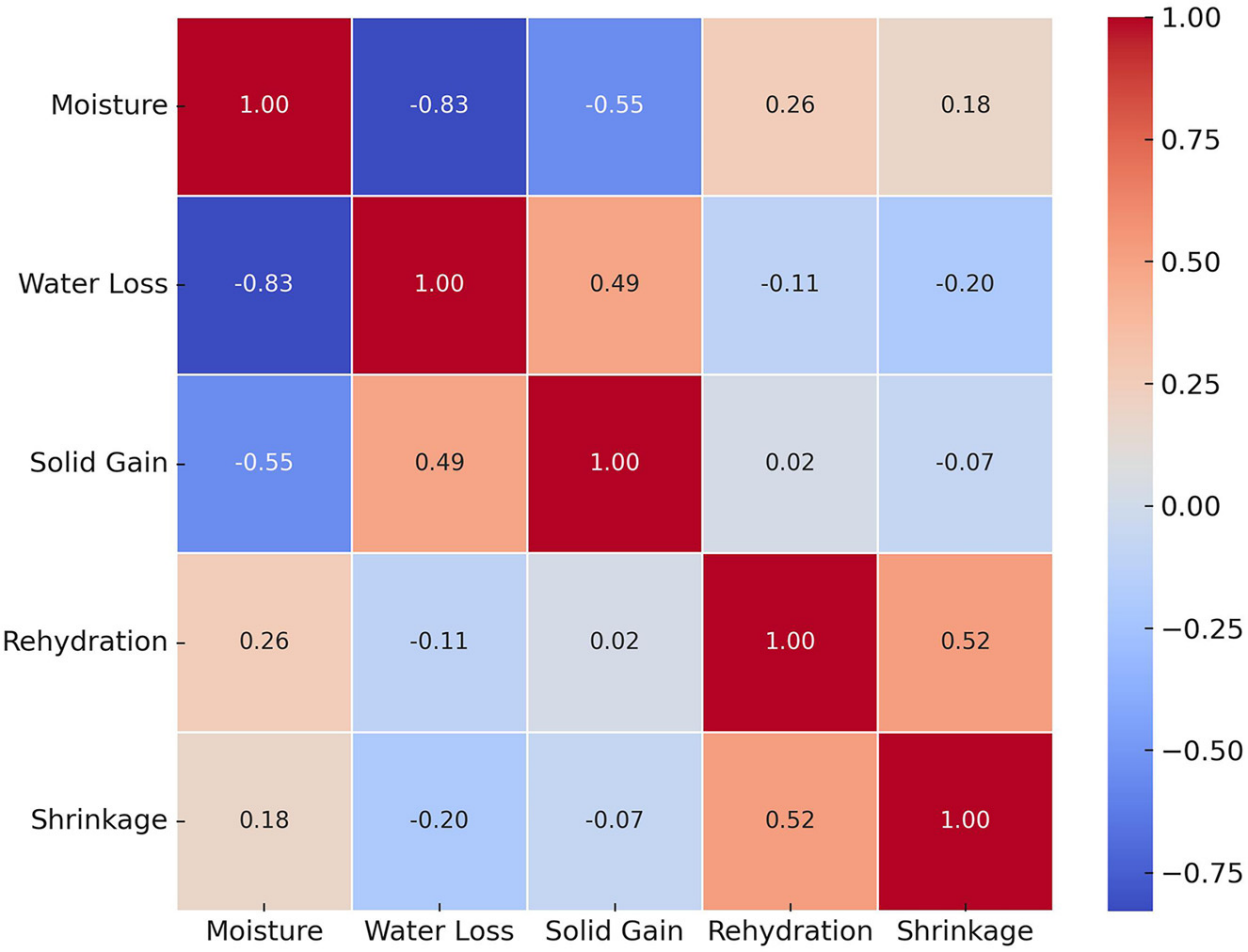
Pearson correlation matrix among technological parameters.

The Pearson correlation matrix (Figure 4) presents a strong negative correlation between moisture content and WL (r = −0.94), and a moderate positive correlation between WL and SG (r = 0.58). These observations align with those reported by Rai et al. [52], who found a strong negative correlation between moisture content and water loss, as well as a positive association between water loss and solid gain in osmotically pre-treated banana slices undergoing hot air drying.

**Figure 5:**
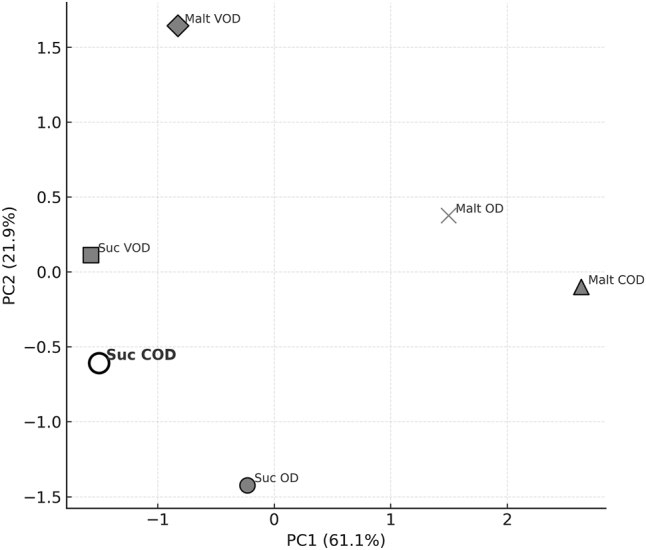
Principal component analysis (PCA) showing multivariate distribution of treatments.

Principal Component Analysis (PCA) was employed to integrate and visualize the multivariate responses associated with the osmotic dehydration treatments, enabling the identification of global patterns and the treatment condition with superior technological performance (Figure 5).

The first two principal components explained 83.0 % of the total variance, with PC1 accounting for 61.1 % and PC2 for 21.9 % of data variability. This high cumulative variance indicates that the reduced two-dimensional space retains the essential structure of the multivariate dataset, enabling a robust interpretation of treatment clustering.

PC1 was predominantly associated with dehydration efficiency, with strong contributions from WL and SG. Treatments with high positive scores on PC1 exhibited greater mass transfer intensity, indicating conditions that promote efficient moisture removal while facilitating controlled solute incorporation. PC2, in turn, was primarily influenced by shrinkage and rehydration capacity, separating treatments based on their ability to preserve structural integrity during osmotic processing. Positive PC2 scores were associated with higher rehydration and lower structural collapse, while negative values indicated more pronounced deformation (Figure 5).

Within this biplot structure, treatments using sucrose (Suc) were clearly distinguished from those employing maltodextrin (Malt), confirming the decisive influence of solute type on overall performance. Sucrose-based treatments consistently occupied regions associated with enhanced mass transfer, whereas maltodextrin treatments clustered in domains indicative of lower dehydration efficacy and more limited solute diffusion – an expected outcome given its higher molar mass and increased solution viscosity [53].

Among all evaluated conditions, Suc COD emerged as the most effective, exhibiting the highest PC1 scores and intermediate PC2 values, which reflect strong dehydration efficiency without excessive structural collapse. This placement indicates that Suc COD simultaneously maximized WL and SG – without inducing excessive structural damage – yielding a technologically desirable balance between efficiency and physical quality. The mechanical action during centrifugal force application likely reduced external resistance to water transfer and promoted intensified dehydration, while preserving enough cellular integrity to ensure satisfactory rehydration behavior.

The multivariate separation of Suc COD from the remaining treatments corroborates the univariate findings and reinforces the synergistic effect of sucrose, elevated temperature, and centrifugal force. Collectively, these factors produced a condition that integrates rapid mass transfer, moderate dimensional stability, and favorable rehydration, confirming Suc COD as the most promising strategy for enhancing osmotic dehydration performance of bocaiuva slices.

PCA has been widely used to interpret complex interactions in OD processes. For example, it has been applied to elucidate the relationships among WL, SG, moisture content, Brix, and water activity in white mushrooms [54].

## 4 Conclusions

The osmotic dehydration of bocaiuva slices was significantly influenced by the type of osmotic agent, temperature, and the application of process intensification techniques. Treatments conducted at 60 °C using sucrose combined with centrifugal force (COD) provided the most efficient moisture removal, greater solute incorporation, moderate shrinkage, and consistent rehydration performance. Multivariate analysis confirmed the synergistic effect of these variables, highlighting the combination of COD with sucrose as the most promising condition for enhancing dehydration performance while preserving product quality.
